# Father–Child Interactions in Preschool Children with ASD: A Systematic Review

**DOI:** 10.3390/brainsci11091202

**Published:** 2021-09-12

**Authors:** Silvia Perzolli, Arianna Bentenuto, Giulio Bertamini, Simona de Falco, Paola Venuti

**Affiliations:** 1 Laboratory of Observation, Diagnosis and Education (ODFLab), Department of Psychology and Cognitive Science, University of Trento, 38122 Trento, Italy; arianna.bentenuto@unitn.it (A.B.); giulio.bertamini@unitn.it (G.B.); simona.defalco@unitn.it (S.d.F.); paola.venuti@unitn.it (P.V.); 2 Data Science for Health (DSH), Bruno Kessler Foundation (FNK), 38123 Povo, Italy

**Keywords:** autism spectrum disorder (ASD), fathers, father–child interaction, paternal involvement, ASD intervention

## Abstract

Studies on parental interaction in the context of ASD has mainly focused on mothers, even if fathers and their children seem to form close and supportive relationships that may have unique effects on child development. Given the impact of ASD symptoms on a child’s ability to interact with significant others, recent findings strengthen the importance of including caregivers during treatment to guarantee a better adaptation to the child’s impairments. Despite this, fathers are scarcely involved, and interventions seem to not be tailored to their interactive characteristics and needs. For this reason, a systematic review was conducted to investigate fathers and children with ASD behaviors during interaction. This review found 12 observational studies that identified social, cognitive, and affective interactive modalities in father–child dyads through three psychology-focused journal databases: PubMed, PsycINFO and Scopus. The significant variation in both sample size and in the measures used to assess dyadic outcomes limits the ability of this work to make robust recommendations for intervention. Despite this, the results revealed characteristic behaviors of this dyad that consequently allow specific targets to be worked on during intervention. In fact, from fathers’ individual strengths and weaknesses, it is possible to implement interventions that are complementary with maternal characteristics from the perspective of personalized and optimized treatment.

## 1. Introduction

### 1.1. The Current Perspective of Fatherhood

It is well established that favorable caregiving experiences have a positive impact on a child’s cognitive development [1,2]. Traditionally, studies on human parenting have focused more extensively on mothers, the most common primary caregivers, than on fathers, leaving fathering still under-investigated. In fact, most research has focused on the strengths and weaknesses of maternal interactive style, as well as on the long-term benefits of mother–child relationships [3,4,5]. However, society’s changes over the past 60 years have contributed to the reshaping of the traditional division of labor in the family, leading to fathers being more involved in parenting and childcare. In line with this, in recent decades, there has been great interest in understanding fathers’ quantity, quality and patterns of interaction with their children, as well as in their influence on child development [6,7,8,9,10]. The innovative studies on fathering that followed this contemporary interest highlight the importance of paternal influence on children’s development, beginning even before birth and continuing all over their life [11].

### 1.2. Father–Child Dyads in the Context of Autism Spectrum Disorder (ASD)

In light of the findings concerning typical development, in the last few decades, research has also focused on fathers who are experiencing the development of children with complex neurodevelopmental disorders, such as autism spectrum disorder (ASD). ASD is characterized by persistent and pervasive difficulties in social communication and interaction domains, together with a pattern of restrictive and repetitive behaviors and activities [12]. Core symptoms of ASD dramatically impact on a child’s ability to interact with significant others, including parents, inducing maladaptive caregiver–child interactive circuits that need to be restored to guarantee effective emotional exchanges [13,14]. The marked difficulties in social communication and responsiveness of children with ASD might create a potential barrier for parents to adequately care for their children [15]. For this, parental involvement during intervention seems to be extremely important to guarantee a better adaptation to children’s difficulties and impairments. Most studies on parental interaction with children with ASD focused on mothers, even if fathers and children with ASD seemed to form close and supportive relationships that may have unique effects on children development and emotional regulation [16,17]. Some research showed that mothers and fathers typically exhibit different interaction behaviors and communication strategies with their children [18]. In turn, children seem to engage differently with the two caregivers [19]. Recent findings suggest that fathers are as competent as mothers in engaging their children in interactions [20], even if mothers reported that they engaged in more social behaviors compared to fathers [21]. Fathers seem to also be as skilled as mothers in using simple and complex regulation facilitation tactics and children with ASD turn to fathers as readily as to mothers for assistance [22,23]. Other results also showed how paternal strategies used to support the child’s activities had important effects on reducing child externalizing problems [24]. Finally, fathers seem to give important contributions to the symbolic play of children with ASD [10]. In line with this, the authors in [19] documented a significant association between child language skills and the verbal responsiveness of fathers. Despite the direct role of fathers on the development of children with ASD, fathers, mothers and the family functioning seem to also benefit when general caregiving responsibilities are equally distributed [25]. In fact, greater paternal involvement in childcare was related to fewer depressive symptoms in mothers [26] and more satisfaction in both mothers and fathers [27].

### 1.3. Paternal Involvement in Early Intervention

Given the influence of parents’ qualities and dyadic characteristics on child developmental outcomes, recent findings strengthened the importance of involving caregivers during the intervention to increase dyadic levels of syntonization and to extend the acquisition of competencies also in naturalistic contexts (e.g., home) [28,29,30]. In fact, better caregiver–child mutual attunement is associated with long-term symptom reduction [31], and to a better generalization of the outcomes across different settings [32]. Interestingly, recent evidence has been found of a significant relationship between the degree of change in parental interaction and the rate of the child’s improvement, underlining the importance of the dyadic relational aspects for child developmental outcomes [33]. Based on the abovementioned results, the study of similarities and differences in interactive behaviors of mothers and fathers towards their children, together with the relevance of these actions on children development, has important clinical implications. In fact, it is fundamental to design optimal interventions tailored to the individual characteristics of both the child and the caregivers [10]. Despite this, recent reviews indicate that fathers are under-represented in empirical investigations of child psychopathology [34,35], even if more positive outcomes are found when both caregivers are included in the therapeutic setting [36]. In their review [10], the authors identified only three studies that specified fathers as being involved in parent training. Yet, especially for children with communicative impairments such as ASD, understanding and enhancing the role of fathers may be an important direction in both research and clinical practice for maximization of the social/communicative gains of children. Realistically, fathers are scarcely involved, even if they desire to be highly involved in their children’s care [37], and they believe that being involved with their children’s education is important [38]. An additional recent systematic review identified 10 studies that included fathers in the context of intervention with different modalities (e.g., in-home intervention, parent groups and workshops, telemedicine, and family intervention) [17]. Even if limited, research suggests an added benefit of having a second parent present during training [17]. Further, in the past few decades, the very few studies investigating the benefits of involving fathers in the treatment of their children with ASD [10], although consistently reporting positive outcomes with respect to the child, did not always find an improvement in the quality of fathers’ behavior [39,40]. In addition, significantly greater increases in self-efficacy have been reported for mothers compared to fathers [41]. Consequently, it has been hypothesized that the support for families of children with ASD may not be tailored to the specific characteristics and needs of fathers [42]. Indeed, due to the emphasis historically put on the study of interactive modalities of mothers, the techniques implemented by therapists during intervention might be more focused on maternal characteristics and interaction styles (e.g., play) than on paternal ones. In fact, having both parents involved may improve the overall family system across many levels. For this, fathers could be trained to be as effective as mothers and, even more interestingly, they may provide unique and still scarcely explored benefits to children with ASD depending on their interaction modalities.

### 1.4. The Current Work

A key element for an accurate and adequate adaptation of fathers during intervention should be based on theoretically grounded principles specifically relevant to fathering behaviors. Despite the important findings mentioned so far, to our knowledge, there is still little evidence on the interaction skills of fathers in social exchanges with their children, often with inconsistent and non-systematic results. However, by developing a better understanding of the behavioral commonalities and differences between fathers and mothers, and among fathers of children with different clinical conditions, therapists may be able to provide parent-tailored evidence-informed training that maximizes each parent’s strengths and the child’s behavioral tendencies. With this in mind, the current work aims to:

- Systematically identify studies considering the social, cognitive, and affective interaction modalities of fathers in interaction with their preschool children with ASD;
- Compare fathers’ and mothers’ interactive behaviors;
- Describe fathers of children with ASD in comparison with different conditions (e.g., typical development, Down syndrome);
- Explore and summarize paternal characteristics that might have relevant implications for the clinical practice;
- Highlight under-investigated areas in the literature to orient future directions.

From a clinical standpoint, understanding the role of fathers may allow both researchers and clinicians to implement parental-based interventions, also taking into consideration particular paternal interactive qualities. Moreover, from a theoretical point of view, this work may be relevant for developing a comprehensive view on father–child interaction while, at the same time, pointing out possible gaps in the literature that should be filled to respond to the contemporary pressing interest on the role of fathers in the development of children with ASD.

## 2. Materials and Methods

The guidelines for the Preferred Reporting Items for Systematic Reviews and Meta-Analysis (PRISMA, ref. [43]) were used. All data of the included studies were anonymized. There is no community involved in this study. The review methods considering the review question, the search strategy and the inclusion and exclusion criteria were established prior to conducting the review. Following PRISMA principles, a formal risk of bias assessment of included articles was considered. However, given the heterogeneity and complexity of the included studies, the decision was not to proceed with a formal assessment as there was not a single quality measure able to capture all these aspects. For this reason, the quality of the studies was evaluated by the authors in terms of sample size, comparison group/s and operationalization of tools to measure observative behaviors (Table 1).

### 2.1. Article Search and Selection

The current study examined articles published in peer-reviewed journals with no restrictions on publication year, up to and including 2020. Articles were collected through three psychology-focused journal databases: PubMed, PsycINFO and Scopus. Three groups of keywords were used interchangeably in different searches of articles related to father–child interactions. All possible combinations included one of the following considering fathers: “father”, “fathers” or “paternal”; one of the following keywords considering autism: “autism”, “ASD” or “autism spectrum disorder”; and one of the following related to interaction: “relationship”, “interaction”, “play”, “responsiveness”, “synchrony”, “attunement”, “structuring”.

Different combinations used each different word (i.e., father AND autism AND interaction or fathers AND ASD AND play) in logical conjunction. PubMed reported 209 articles, PsycINFO reported 253 articles and Scopus reported 378 papers considering the combinations of chosen keywords (*n* = 840). In addition to this, one article was manually added for a total amount of 841. Data extraction was based on the recommendations of the “Cochrane Handbook for Systematic Reviews” [44], considering the following information: (a) general information of the study (e.g., authors cited, country of origin); (b) methodology (e.g., clear design, follow-up); (c) sample (e.g., sample size, recruitment, demographics); (d) results (e.g., effects that are found); (e) further information (e.g., statistical information, effect sizes).

### 2.2. Inclusion Criteria—Title/Abstract

During the title and abstract review process, the articles were included in this work only if they met the following inclusion criteria:

1. Peer-reviewed articles (case studies, editorial pieces, meta-analyses, and systematic reviews were not included in the results of this review). We wanted to include works with high power and relevance and, therefore, we did not consider case studies nor editorials.
2. Papers should be printed in English.
3. A diagnosis of ASD conducted through standardized instruments (e.g., ADOS, ADI) following the DSM criteria and clinical observations.
4. Children should be in an age range from 2 to 6 years.
5. Fathers of children with ASD should be part of the sample.

Duplicates resulting from the search in different databases were removed. At this stage, articles that referred to developmental delays with no ASD diagnosis were excluded from the search. The screening process excluded 816 papers from the initial research (*n* = 355 were excluded for duplicates and *n* = 461 were excluded due to the abstract). Therefore, *n* = 25 papers passed the first screening.

### 2.3. Inclusion Criteria—Article Review

For this process, we carefully read the entire papers to identify whether the materials should be considered further in this review. Two reviewers extracted data from a sample of eligible studies and achieved very good agreement (96%), with the remainder extracted by one additional reviewer. When the two reviewers disagreed, another author’s opinion was sought. The same reviewers collected data from each report independently. We included articles that considered the following inclusion criteria:

1. To ensure that the results of the study specifically referred to fathers, they must have been mentioned in the Methods part, where the authors describe the participants, and in the Results section. In fact, the current work excluded studies that showed: (1) Results of fathers not distinguished from the ones of mothers (e.g., if they were grouped together as “couples” or “parents”); (2) Results of fathers not distinguished from the ones of fathers of children with other developmental delays or disorders. In line with this, when preschool children with ASD were part of the sample, but results were discussed considering autism together with other clinical conditions such as “neurodevelopmental disorders”, “developmental delays” or others, these studies were also excluded from the review.
2. Observational studies in which father–child behaviors were investigated. Coherently with the aims of this review, we wanted to systematically collect the behavioral interaction elements of fathers while interacting with their preschool children with ASD. Consequently, it was necessary to look for studies that provided interactive sessions between the dyad and that provided coding for informative outcomes. Indirect measures considering dyadic aspects are not included (e.g., self-report questionnaires).
3. Sufficient statistical information should be reported. For an appropriate interpretation of the results, some statistical information should be reported in the papers (means, standard deviations, detailed description of the conducted analysis).

**Figure 1:**
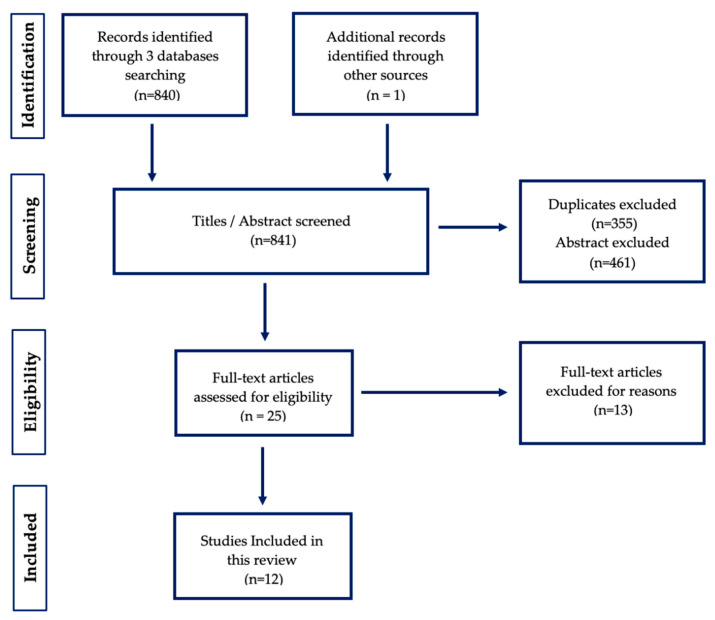
PRISMA diagram for interactive behaviors article search.

At this point, 25 articles were screened for eligibility and 12 articles passed the criteria (Figure 1).

### 2.4. Excluded Studies

In this section, we describe the 13 potentially relevant studies out of the 25 that passed the first screen, which were read in full-text form but excluded from the review for not meeting the inclusion criteria described above. A justification for exclusion from the review was given for each study.

Specifically, *n* = 7 studies were removed from the search because the sample size did not meet the inclusion criteria. Four papers were eliminated because of the age of the children with ASD, ranging from 4 to 10 years [45] or from 5 to 12 years [46,47,48]. In addition to this, another work [49] was not considered further because the 12 participants with ASD were in the age range between 3 years and 8 months to 11 years and 8 months. Another work [50] was excluded because the sample of 38 children included a wide range of clinical conditions and the results were discussed in terms of young children with “special needs”. For this, it was impossible to disentangle the specific role of children with ASD in father–child relationships. Further, the age of the sample was from 6 to 34 months. In addition to this, another recent work was deleted [51]. Even though this paper considered a sample of 24 fathers and 26 mothers, results were discussed considering the two caregivers togethers as “parents”. Another three studies [52,53,54] were not considered further because the sample consisted of children “at-risk” of developing ASD. Another work [55] regarded only one father considering the sample of children with ASD, and therefore, was no longer considered. Finally, another study [56] was excluded because the sample mainly considered mothers except for two fathers.

## 3. Results

From the search in PubMed, four works met the inclusion criteria established for this review. PsycINFO revealed three new studies that were included in the work. Finally, Scopus added four more studies that were included in this study. Furthermore, one more study was manually added to the review [18]. A total number of *n* = 12 studies met the eligible criteria.

### 3.1. Sample Demographics

#### 3.1.1. Child Age, Gender and Ethnicity

The samples of the current review considered children in the preschool range of age. Three studies considered children from 24 to 71 months of age [57,58,59]. One study took into account an age range from 32 to 76 months [60]. Two studies involved children aged from 36 to 82 months [16,61]. Additionally, three studies included children from 38 to 73 months [62], 38 to 88 months [63] and 40 to 68 months [18], respectively. Finally, three studies took into consideration children from 40 to 69 months [10,19,64]. Table 2 presents demographic information.

All studies apart from two [59,63] reported information about the gender of the children with ASD. The samples were largely composed of males in all studies, except [18], in which only 48.2% of children with ASD were males. In particular, five studies [10,19,58,60,64] appeared to be consistent with the updated estimates of males being four times more likely than females to be diagnosed with ASD [65] (Table 2).

Most of the studies reported information about the ethnicity of participants. One study considered an Italian sample [57] and two studies collected data from the Turkish population [18,60]. Moreover, two studies analyzed the American population [62,63] and three additional studies considered a mixed population made up of white/non-Hispanic, Hispanic, and Asian (Table 2).

#### 3.1.2. Control Group/s

A large majority of the studies considered in this systematic review include a control group. N = 7 studies referred to the comparison between fathers and mothers in interaction with their preschool children [10,19,57,59,62,63,64]. Other studies took into consideration both fathers and mothers of children with ASD and of children with typical development [16,61]. One study considered fathers of children with ASD compared to fathers of children with Down syndrome [18]. Finally, one study included fathers of children with ASD in comparison with fathers of children with typical development and Down syndrome [58].

### 3.2. Observational Studies

All the studies included in this systematic review are observational and behaviors of fathers in interactions with their preschool children with ASD were observed. Despite this, there are differences in the modalities through which fathers were asked to interact with their children, especially considering:

- Context;
- Time;
- Toys.

#### 3.2.1. Context

The observed interactions between fathers and their children included different contexts. In most studies, fathers could play with their children in a laboratory setting [10,18,19,57,58,59,60,61,64]. However, one study took into consideration a more naturalistic setting such as the home environment [62]. Other studies, indeed, considered more than one setting. Other authors [63] provided observations from both the clinic playroom and the child’s home. Further, in this work [16], behaviors of fathers and children were coded during emotion regulation (ER) paradigms, eliciting both negative and positive emotions.

#### 3.2.2. Time

Among the studies, interactions differed in the amount of time spent in dyadic play. One study analyzed video-recorded interactions for 25 min [63]. Instead, some studies considered 15–20 min of play interactions [10,18,19,58,60,62,64]. Furthermore, other works used 10 min play sessions [57,61] and another two studies comprised 7 min interactions [16,59].

#### 3.2.3. Toys

In the analysis of interactive behaviors of the father and the child with ASD, there are also differences considering toys. In fact, some studies did not provide any toys and asked parents to spontaneously play with the child with no objects [60]. In one study, toys were not standardized. Instead, they were spontaneously chosen by the family from a set of toys that the parents chose to dispose of in the playroom [62]. Other studies used standardized sets of toys furnished by the experimenter based on age [10,18,19,57,58,61,63,64]. Finally, two studies used standardized toys focused to elicit symbolic play [16,59].

### 3.3. Dyadic Behavioral Outcomes

Studies analyzed in this systematic review can be divided into two categories of outcomes: *emotional and affective behaviors* and *cognitive behaviors* that include play and linguistic abilities.

#### 3.3.1. Emotional-Affective Behaviors

Considering the first domain, one study [60] revealed that even if fathers exhibited a moderate to low level of interactive behaviors with their children with ASD, increasing levels of paternal sensitivity and responsiveness were correlated to greater child engagement. On the contrary, when fathers assumed a controlling role, child engagement levels tended to decrease. Other authors [57] found that, although not optimal, fathers seem to show moderate to good levels of sensitivity, revealing no differences between mothers’ and fathers’ scores. Additionally, previous studies [63] identified no differences between the two caregivers in their frequencies of initiating and responding during the interactions with their children. However, some evidence showed that fathers of children with ASD scored lower on responsiveness, affect, attention and initiation with respect to mothers [18]. In fact, other authors [61] also found that fathers showed less support and less involvement than mothers in interaction with their children with ASD. Further, when the context was negative for the children (e.g., fear context), they seemed to show equal levels of assistance-seeking behaviors towards mothers and fathers [16]. Results also appear to be controversial considering the child’s point of view. In fact, some research pointed out that children seemed to respond and involve the caregivers equally [57,63]. However, other findings underlined differences in child’s engagement with mothers and fathers [16]. Finally, considering this domain of behaviors, an interesting study [59] shed light on the idea that father–child reciprocal behaviors may have an impact on stress levels. In fact, when father–child dyads showed more coordinated and better dyadic functioning, they also showed less correlated levels of cortisol. For a detailed description of the measures and study outcomes of the included studies, see Table 3.

#### 3.3.2. Play and Linguistic Behaviors

With respect to other characteristics of fathers in interaction with their children with ASD, research mainly focused on play and linguistic behaviors.

Considering play situations, fathers of children with ASD looked at their children more frequently than fathers of TD. They also seemed to engage in more physical contact with the child compared to fathers of children with Down syndrome (DS) and typical development (TD). These fathers also seemed to display higher frequencies of direct play proposals addressed to the child compared to fathers of children with TD. In turn, children seemed to respond with more relational play when they were with their mothers compared to father–child interactions [10].

Considering linguistic aspects of fathers of children with ASD, the reviewed literature suggests that fathers generally displayed less statements than mothers when interacting with their children. Nonetheless, their children seemed to attempt more vocal/verbal initiations towards them when compared to their mothers [62]. In line with this, other findings suggest that fathers seemed to use a lower frequency and proportion of verbal responses [19]. Finally, more recent research investigated specific characteristics of the broader autism phenotype such as aloofness and rigidity. These characteristics were found to be unrelated to the levels of the child’s language skills and child-initiated behaviors. Further, paternal pragmatic deficits were also found to be not associated with the child’s language, but positive correlations were found between paternal pragmatic difficulties and the frequency of child-initiated engagement [64]. These findings seem to suggest that children in the sample used more engagement tactics with fathers whose pragmatic language characteristics were similar to the ones of individuals with ASD, even if this warrants further investigation.

## 4. Discussion

In the last few years, fathers have gained importance in the context of both typical [1,6] and atypical development [2,17]. Moreover, the role of fathers significantly evolved due to societal changes and, consequently, their presence in childcare greatly increased. Recent literature also highlights the importance of involving parents during interventions in the context of ASD [29,33,66]. However, the vast majority of these studies were conducted on mothers, and therefore, interventions are often tailored without considering father’s behaviors, characteristics, and needs [42]. Despite being underrepresented in the literature, research on fathers seems to show similarities but also differences with respect to mothers when interacting with their preschool children, and these particular interactive modalities need further investigation. Considering this, the purpose of this review was to systematically outline father–child interactive patterns in the context of ASD in preschool children. Although important studies have been conducted so far, they also present several limitations that prevent depiction of a clear and coherent profile of fathers, undermining the generalizability of the results. However, understanding the role of fathers may have important clinical implications for ASD treatment, especially considering the empirical support recently obtained by parent-mediated interventions [67,68,69,70]. The studies found in the literature and included in this review seem to be highly heterogeneous in their methodological approach. In fact, the sample size of the studies ranged from 6 to 40 participants including mostly males, coherent with the estimated prevalence of ASD [65]. This variability in the sample size underlines how effortful it is to conduct studies on this specific population. In addition to this, the works analyzed so far also refer to different cultures, making it even more difficult to generalize results, considering how much cultural aspects influence parenting styles and behaviors [3,71].

Another important aspect of the studies considered in this review regards the different observational settings and their structuring level in terms of duration, set of toys and verbal indications given to the parents. In fact, the different duration of the interactions might encourage different behaviors in the dyadic context with different probabilities to be observed. In the same way, the set of toys available during the observations may obstruct or promote the dyadic exchange and play in different ways. Further, conducting observational studies in different environments, from freer to more structured ones, influences the interactive modalities both directly and indirectly. Consequently, these results are difficult to measure in terms of external validity.

An additional issue concerning the studies in this review is the use of heterogeneous measures to collect data considering interactive behaviors between fathers and their children with ASD. In fact, affective exchanges and dyadic play are measured through different standardized or non-standardized tools that refer to different metrics and operationalizations, making it difficult to compare the studies. Heterogeneity in the methodological approach can also be noticed in the results, which appear to be inconsistent in the definition of father–child interaction profiles. The variability in the methods and the heterogeneity of the results seem to pose a challenge to a unitary vision of paternal characteristics in the context of ASD.

In line with this, some studies revealed no differences in engagement and responsiveness between fathers and mothers [57,63]. In other studies, fathers showed lower scores in these domains when compared to mothers [18,60]. However, their levels are still positive and, therefore, their presence should be considered relevant for child’s development. The different results may also be at least partly explained by the different cultural contexts in which the studies are conducted [3,71].

From the child’s point of view, the results are still controversial with respect to interactive modalities towards mothers and fathers. However, findings seem to be more homogeneous when children with ASD are compared to children with TD or Down Syndrome (DS). In fact, the core impairments of children with ASD in the socio-interactive domain dramatically impact on parental abilities [13,14].

In addition to this, the analysis of play and linguistic characteristics of fathers in interaction with their children with ASD highlighted different profiles when comparing both mothers and fathers and children with different clinical conditions. Fathers of children with ASD seemed to show a more controlling modality during the interchange, when compared to fathers of children with TD or DS [58]. However, this particular element does not emerge in the comparison between mothers and fathers with their children with different disabilities, including autism. In fact, in this case, mothers seem to show more controlling and intrusive behaviors both when playing and speaking to the child [72]. Taken together, these findings seem to suggest that fathers are characterized by a greater amount of social and physical engagement [58], while mothers seem to use more structured and more direct support strategies (e.g., language) to interact with the child [61]. Furthermore, considering child emotional regulation, some results highlighted differences in child seeking behaviors and regulation strategies towards fathers during emotionally positive situations. Interestingly, no differences were found in child seeking behaviors during emotionally negative situations [16], highlighting the importance of both parents as a secure base for the child.

Considering the domains of language and play, some elements seem to characterize the father–child dyad. Even if fathers seem to display less statements and less proportion of language when interacting with their children compared to mothers, both parents’ verbal behaviors seem to be associated with more sophisticated levels of child’s play [10]. In addition to this, recent research seems to show that fathers of children with ASD display a lower proportion of questions to the child and a greater proportion of descriptions and verbalizations compared to fathers of children with TD [73]. This finding suggests that fathers may use high levels of verbal structuring in favor of a reduction in requests. Conversely, mothers of children with ASD seem to show linguistic features characterized by a more controlling style when compared to mothers of children with TD and DS [74]. Both similarities and differences between parents seem to represent important aspects that need to be integrated to support the development of child’s abilities. With this in mind, the paternal and maternal role might be seen in a complementary way to guarantee an adequate and harmonious development of the child.

The evidence discussed so far may also be relevant in the context of ASD parental-based interventions. In fact, despite several studies suggesting the fundamental role of caregivers during intervention, these results mainly refer to mothers. For this, interventions are designed based on evidence that may not be generalized to fathers. Therefore, given the paucity of research conducted so far, this review highlights both the importance of conducting research focusing on fathers in the context of ASD and the need for more systematic designs and more representative samples in studies on parenting, specifically in the context of preschool children with ASD. These efforts may also be relevant in the perspective of personalized and optimized parental-based interventions tailored not only to the single child, but also to the specific dynamics that characterize father–child and mother–child interactions [75]. In fact, some studies highlighted longitudinal changes in dyadic patterns [63,76,77], suggesting that working on father–child interactive modalities may also have an impact on child’s behaviors and reciprocal attunement. Considering both similarities and differences between parents may be relevant for personalized interventions, in order to identify optimal interactive strategies with the child that may differ between them. Finally, the two parents may differentially work on specific targets of the intervention based on their own individual characteristics and strengths, from the perspective of optimized treatment.

Despite the relevance of this work in identifying fathers’ behaviors in interaction with their preschool children with ASD, there are limitations of the review process to discuss. Considering the methodological approach to conducting systematic reviews, we did not use a satisfactory technique for assessing the risk of bias (RoB) in all the studies given the heterogeneity of the methodological approaches. Further, we did not include grey literature in the search, preventing the possibility to look for additional information considering the topic. Additional limitations that prevent the generalization of the findings deal with the fact that the results were not always detected through standardized observational tools. Finally, information about the amount of time the caregivers spent with the child was rarely provided and, therefore, it was difficult to disentangle if the results were due to the “parental role” or to the fact that they were considered the *main* caregiver of the child.

### Future Research

From the analysis conducted so far, future works should include studies with a higher sample size for better generalization of the results. Moreover, including longitudinal designs might allow for the investigation of both child behaviors and variables (e.g., cognitive functioning and symptoms severity) and paternal traits that might have an impact on interaction quality specifically and, in turn, on child development. Further, it would be important to include standardized instruments and quantitative variables such as the amount of time spent with the child by the two caregivers.

## 5. Conclusions

To conclude, it is worth noticing that in the next few years, ongoing societal and cultural changes will emphasize the evolution of the role of parents in childcare and development even more. Fathers will increasingly be involved in all aspects of parenting and eventually shape their own ways of rearing children in synergic complementarity with the maternal role. Such changes further emphasize the current need for improving research on fathers’ involvement in parental-based interventions for children with ASD, as well as its translational implications to inform clinical practice in a research-oriented perspective based on empirical evidence.

## Figures and Tables

**Table 1:** Quality of the included studies in the systematic review.

Source	Sample Size	Control Group/s	Operationalization of Tools
Arslan and Dicken, 2020	*++*	*+*	*+++*
Bentenuto, Perzolli, de Falco and Venuti, 2020	*+++*	*++*	*+++*
El-Ghoroury and Romanczyk, 1999	*+*	*++*	*+*
Elder, Valcante, Won, Zylis, 2003	*++*	*+*	*+*
Flippin and Watson, 2011	*+*	*++*	*+++*
Flippin and Watson, 2015	*+*	*++*	*+++*
Flippin and Watson, 2018	*+*	*++*	*+++*
Hirshler-Guttenberg, Golan, Ostfeld-Etzion and Feldman, 2015	*+++*	*+++*	*+++*
Karaaslan, 2016	*++*	*+++*	*+++*
Ostfeld-Etzion, Feldman, Hirschler-Guttenberg, Laor and Golan, 2016	*+++*	*+++*	*+++*
Pisula 2008	*+*	*+++*	*+*
Saxbe, Golan, Ostfeld-Etzion, Hirschler-Guttnberg, Zagoory-Sharon and Feldman, 2017	*+++*	*++*	*+++*

+ Low quality (small sample size from 9 to 20, no comparison group, no operationalization of tools); ++ Average quality (acceptable sample size from 20 to 30, presence of a comparison group, tools are operationalized); +++ High sample size (from 40 onwards, presence of comparison groups, tools are operationalized and validated).

**Table 2:** Demographic Statistics of the Studies.

Source	N of Children(Per Sample)	N of Parents	Child Age Range and/or Mean (months)	Child Diagnosis
Arslan and Dicken, 2020	30	30	32–76 months	ASD
Bentenuto, Perzolli, de Falco and Venuti, 2020	40	40 fathers; 40 mothers	24–71 months	ASD
El-Ghoroury and Romanczyk, 1999	9	6 fathers; 9 mothers	38–73 months	ASD
Elder, Valcante, Won, Zylis, 2003	22	22 fathers; 22 mothers	38–88 months	ASD
Flippin and Watson, 2011	16	16 fathers; 16 mothers	40–69 months	ASD
Flippin and Watson, 2015	16	16 fathers; 16 mothers	40–69 months	ASD
Flippin and Watson, 2018	16	16 fathers; 16 mothers	40–69 months	ASD
Hirshler-Guttenberg, Golan, Ostfeld-Etzion and Feldman, 2015	39; 40 = 79	39 fathers; 39 mothers in ASD group; 40 fathers; 40 mothers in TD group	36–82 months	ASD—TD
Karaaslan, 2016	27	16 fathers; 16 mothers in ASD group; 11 fathers; 11 mothers in DS group	40–68 months	ASD—DS
Ostfeld-Etzion, Feldman, Hirschler-Guttenberg, Laor and Golan, 2016	39; 40 = 79	39 fathers; 39 mothers in ASD group; 40 fathers; 40 mothers in TD group	36–82 months	ASD—TD
Pisula 2008	14; 15; 16	14 fathers of children with ASD; 14 fathers of children with DS; 14 fathers of children with TD	24–71 months	ASD—DS—TD
Saxbe, Golan, Ostfeld-Etzion, Hirschler-Guttnberg, Zagoory-Sharon and Feldman, 2017	39; 40	39 fathers; 39 mothers in ASD group; 40 fathers; 40 mothers in TD group	24–71 months	ASD

**Table 3:** Study outcomes.

Source	Method	Measures	Dyadic Outcomes
Arslan and Dicken, 2020	Interactions were videotaped for 15–20 min in a free-play context.	■ Interaction behaviors of fathers were analyzed by the Maternal/Paternal Behavior Rating Scale-Turkish Version (Dicken, 2009). ■ Interaction behaviors of children with ASD were analyzed by the Child Behaviors Rating Scale-Turkish Version (Dicken, 2009).	■ Fathers exhibited a moderate to low level of interactional behaviors with their children with ASD. ■ If sensitivity, responsiveness, and emotional expressiveness of fathers increased positively, child engagement increased as well. ■ When fathers used achievement-oriented, directive or teaching styles, child engagement decreased.
Bentenuto, Perzolli, de Falco and Venuti, 2020	Interactions were videotaped for 10 min in a semi-structured context. It was asked that the father spontaneously play with their child as if they were at home with a set standard of toys. Sessions were videotaped in a laboratory setting.	■ Interaction behaviors of mothers and fathers were measured through Emotional Availability Scales (EAS, Biringen et al. 2008) considering adult scales of Sensitivity, Structuring, Non-Intrusiveness and Non-Hostility. ■ Interaction behaviors of children was measured through Emotional Availability Scales (EAS, Biringen et al. 2008) considering child scales of Responsiveness and Involvement. ■ Scores were given after appropriate training by two independent observers.	■ No differences emerged in mothers and fathers’ Emotional Availability towards their children. ■ No differences emerged in child’s level of Responsivity and Involvement when interacting with their mothers and fathers.
El-Ghoroury and Romanczyk, 1999	Play interactions of family members were videotaped for 15 min; toys were not standardized but chosen by the family in a room of the parent’s choice spontaneously. Toys were selected from family’s collection.	■ Videos were scored from specific verbal and motor behaviors exhibited by the family member from Strain’s research. ■ Video were scored from specific social behaviors exhibited by the child. ■ The definitions for the behavior scores were listed and coded by three independent raters, observing only one partner of the dyad at a time.	■ Fathers seem to reveal less statements than mothers when interacting with their children. ■ Children with ASD seem to have more vocal/verbal initiations towards their fathers compared to their mothers.
Elder, Valcante, Won, Zylis, 2003	Each parent–child dyad was video-recorded for 25 min playing with toys together in two settings: a clinic playroom and the child’s home. Toys remained consistent for each dyad. Parents were instructed to play as they normally would with the child.	■ Caregivers were coded considering the following behaviors: caregiver initiations, caregiver responses, caregiver-initiated turns completed. ■ Children were coded considering the following behaviors: child vocalizations, child stereotypes, child intelligible words, child-initiated turns completed.	■ No significant differences between mothers and fathers in their frequencies of initiating and responding during the interactions with their children. ■ Wide variety of initiating and responding rates between individuals of the same gender and between caregivers in the same family. ■ No differences in child’s interaction with each parent nor each context.
Flippin and Watson, 2011	Three 15-min free-play observations (unsupported play with an unfamiliar person, mother–child, and father–child) with standardized, parallel toy sets depending on the situations. Interactions were coded at 5 s intervals using a coding system.	■ Considering caregivers, Parent Play Responsiveness and Parent Play Responsiveness were coded. ■ Considering the child, Child leads (touch, look) and Child Object Play were coded.	■ Children tended to engage in more relational play with mothers than unsupported play or play with fathers. ■ For both, use of responsive verbal behaviors was correlated with higher level object play. ■ Use of responsive play behaviors was correlated with higher level object play only for fathers.
Flippin and Watson, 2015	Mothers and fathers were individually video-recorded in a naturalistic play setting with their child for 15 min. Toys were selected based on child’s age and sex.	■ Differences in behaviors between the caregivers included: ■ Types of lead used by the children (look, touch or no lead); ■ Presence of parent verbal responsiveness.	■ Children used more leads with their mothers compared to fathers. ■ Fathers seem to use a lower frequency and proportion of verbal responses.
Flippin and Watson, 2018	15 min naturalistic parent–child interaction were videotaped and coded at 5 s intervals with two different standard, parallel sets of age-appropriate toys. Coding was conducted by two independent coders.	■ Interactions were examined to investigate associations among: ■ Parental Broad Autism Phenotype (BAP). ■ Parental verbal responsiveness (parent utterances: responses, requests considering the child not objects). ■ Language skills of children. ■ Child engagement (look, touch or no engagement).	■ For fathers, aloofness and rigidity were unrelated to child-initiated engagement and language skills. ■ Positive associations were found between paternal pragmatic language deficits and frequency of child-initiated engagement.
Hirshler-Guttenberg, Golan, Ostfeld-Etzion and Feldman, 2015	Parents and child engaged in 7 min free-play interactions with preselected toys known to elicit symbolic play. Interactions were coded for parent and child behaviors and children engaged in ER paradigms eliciting negative (fear stimuli were presented by the experimenter) and positive (joy, play with puppets for 5 min) emotions with each parent.	■ Coding Interactive Behavior (CIB, Feldman, 1998) was used. ■ Considering caregiver, Parental Sensitivity, Parental Intrusiveness and Parental Limit Setting were used. ■ Considering the child, Child Involvement, Child Withdrawal and Child Compliance were used. ■ Dyadic reciprocity was also coded.	■ Children with ASD expressed less emotionally overall and more negative emotionality during fear with fathers. ■ Children used more simple self-regulatory strategies but comparable levels of assistance-seeking behaviors toward the mother and father in negative contexts. ■ Children seeking parental assistance were comparable for both caregivers.
Karaaslan, 2016	Interactions of mother–child and father–child were video-recorded in a small room equipped with developmentally appropriate toys for 20 min. Video recordings were scored by two independent coders separately.	■ Caregiver behaviors were assessed through the *“Maternal Behavior Rating Scale (MBRS)”* investigating Responsiveness, Affect and Achievement/Directiveness ■ Child Behaviors were assessed through *“Child Behavior Rating Scale (CBRS)”* investigating Attention and Initiation	■ Mothers of children with ASD and DS are more responsive than fathers. ■ Fathers of children with ASD scored lower on responsiveness, affect, attention and initiation. ■ Regardless of whether the child had ASD or DS, both caregivers’ levels of responsiveness was associated with child’s engagement.
Ostfeld-Etzion, Feldman, Hirschler-Guttenberg, Laor and Golan, 2016	Interactions of mother–child and father–child were videotaped during 10 min of free-play with a predefined set of miniature toys provided by the experimenter. Then, the experimenter said, “you have to stop playing now, please pick up the toys into this bag”. The pick-up procedure was videotaped until all toys had been picked up. Videos were microcoded offline for parents’ and child’s behavior on a computerized system by two trained, independent and blind coders.	■ Considering the child, self-regulated compliance, externally monitored compliance and noncompliance were coded. ■ Considering caregivers, harsh control, warm control (direct support, supportive presence), no control (un-involvement, over-involvement).	■ Mothers of children with TD and ASD demonstrated more direct support compared to fathers and less un-involvement.
Pisula 2008	Interactions between fathers and their children were video-recorded for 15-min of free-play context in the experiment room with a set of 20 toys. Interactions were later coded to identify behaviors using a 15 s interval occurrence system by two independent coders.	■ Analysis included 18 behaviors of fathers ■ Analysis included 20 behaviors of children	■ Fathers of children with ASD looked at their children more frequently than fathers of TD. ■ Fathers of children with ASD engaged in more physical contact with their child compared to fathers of children with DS and TD. ■ Fathers of children with ASD had higher frequency of suggesting play to their child than fathers of children with TD. ■ Children with ASD brought objects to their fathers or pointed out objects and directed their fathers’ attention by vocalizing less frequently than children with DS and TD.
Saxbe, Golan, Ostfeld-Etzion, Hirschler-Guttnberg, Zagoory-Sharon and Feldman, 2017	Families of preschoolers participated in two dyadic sessions. Each member of the dyad participated in several interaction tasks that were behaviorally coded and provided three cortisol samples. Parent and child engaged in a 7 min free-play interaction with preselected toys known to elicit symbolic play. Parents were asked to spontaneously play with their child.	■ Interactions were assessed through The Coding Interactive Behavior (CIB, Feldman, 1998). The following factors were used: ■ Parental Sensitivity, Dyadic Reciprocity, Emotion Regulation paradigms, Parent–child Behavioral Coordination, Child Involvement, Child Self-Regulation ■ Cortisol levels were also analyzed	■ Associations between parent–child behaviors and HPA axis linkage revealed that: ■ Father–child linkage was weaker if the father–child dyad showed more reciprocity and more sensitive father behavior, as well as more child self-regulation and less child withdrawal. ■ When the father–child dyad showed more coordinated and better dyadic functioning, they had less correlated levels of cortisol across their home visit.
